# Caseating Granulomas in Cutaneous Leishmaniasis

**DOI:** 10.1371/journal.pntd.0003255

**Published:** 2014-10-23

**Authors:** Jessica Aoun, Robert Habib, Khalil Charaffeddine, Suad Taraif, Asif Loya, Ibrahim Khalifeh

**Affiliations:** 1 Department of Pathology and Laboratory Medicine, American University of Beirut Medical Center, Beirut, Lebanon; 2 Department of Internal Medicine, Outcomes Research Unit, American University of Beirut Medical Center, Beirut, Lebanon; 3 Department of Pathology, Saad Specialist Hospital, Al Khobar, Kingdom of Saudi Arabia; 4 Department of Pathology, Shaukat Khanum Memorial Cancer Hospital and Research Centre, Lahore, Pakistan; Emory University, United States of America

## Abstract

**Background:**

Caseating granulomas are often associated with a mycobacterial infection (TB) and are thought to be exceedingly rare in cutaneous leishmaniasis (CL). However, no large series has accurately documented the incidence of caseating granulomas in CL.

**Methods:**

A multiregional cohort consisting of 317 patients with CL [Syria (157), Pakistan (66), Lebanon (47), Saudi Arabia (43), Ethiopia (2) and Iran (2)] was reviewed. Clinical [age, sex, disease duration, lesion type and geographic and anatomic location] and microscopic data [presence of and type of granuloma, Ridley's parasitic index (PI) and pattern (RP)] were documented. Presence of microorganisms was evaluated using special stains (GMS, PAS, AFB and Gram) and polymerase chain reaction (PCR) for TB and CL. All cases included in this study were confirmed as CL by PCR followed by restriction fragment length polymorphism analysis for molecular speciation and were negative for other organisms by all other studies performed. Categorical and continuous factors were compared for granuloma types using Chi-square, t-test or Mann-Whitney test as appropriate.

**Results:**

Granulomas were identified in 195 (61.5%) cases of CL and these were divided to 49 caseating (25.2%), 9 suppurative (4.6%) and 137 tuberculoid without necrosis (70.2%). Caseating and tuberculoid granuloma groups were significantly different in terms of the geographical source, with more cases harboring caseating granulomas in Saudi Arabia (p<0.0001). Histologically, both groups were also different in the distribution of their RP (p<0.0001) with a doubling RP3 in caseating granulomas (31% vs. 15%) as opposed to doubling of RP5 in tuberculoid granuloma group (38% vs. 19%). Time needed to achieve healing (RP5) was notably shorter in tuberculoid vs. caseating group (4.0 vs. 6.2 months). Parasitic Index, CL species and other considered variables did not differ for the granuloma type groups.

**Conclusion:**

In our multiregional large cohort, a notable 18.2% of all CL cases harbored caseating granulomas therefore; CL should be considered part of the differential diagnosis for cases with caseating granulomas in endemic regions, especially considering that the regions included in our cohort are also endemic for TB. Of note, cases of CL with caseating granulomas also showed a slower healing process, with no association with specific species, which may be due to worse host immune response in such cases or to a more aggressive leishmania strains.

## Author Summary

Cutaneous leishmaniasis displays a wide spectrum of clinical and microscopic findings. The microscopic manifestations have been categorized into five groups. The type of granulomatous response defined in group V is usually tuberculoid in nature with exceedingly rare cases described with necrotizing granulomas in contrast to cutaneous infections with tuberculosis and other mycobacteria that are typically associated with necrotic granulomas. The old world countries endemic for cutaneous leishmania also happen to be endemic for other granulomatous diseases such as leprosy, tuberculosis and cutaneous mycoses. The most common diagnostic approach used in these countries is still microscopic examination despite the advances in molecular diagnostic techniques and culture methods. We document an 18.2% incidence of caseating granulomas in cutaneous leishmania. Hence, cutaneous leishmania should be part of the differential diagnosis for cases with caseating granulomas in endemic regions in addition to other causative infections.

## Introduction

**Figure 1:**
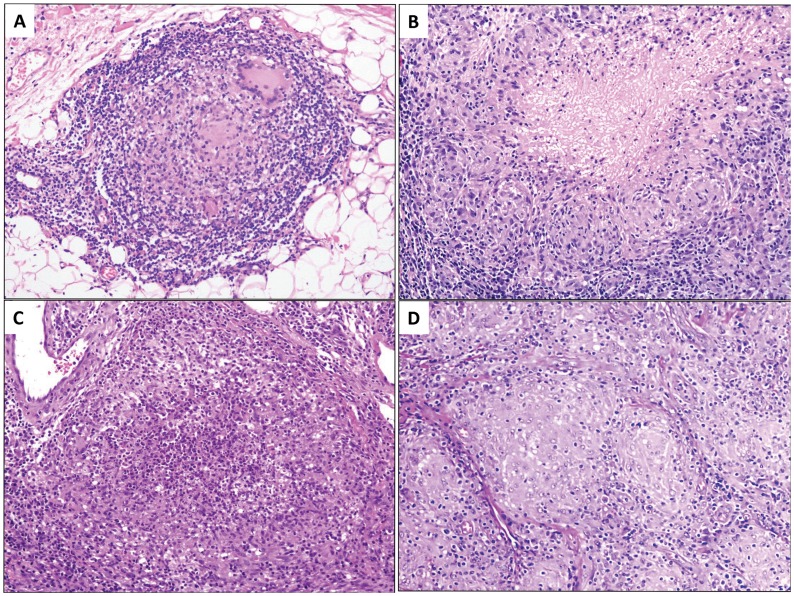
Tuberculoid granuloma with cuff of lymphocytes coating aggregate of epithelioid histiocytes and giant cells without necrosis in the center (A, hematoxylin and eosin stain, mag. 10X). Caseating granuloma with caseating necrosis surrounded by epithelioid histiocytes (B, hematoxylin and eosin stain, mag. 20X). Suppurative granuloma with mixed inflammatory infiltrates and necrotic debris in the center (C, hematoxylin and eosin stain, mag. 20X). Sarcoidal/naked granuloma without cuffing lymphocytes and no necrosis noted in the center (D, hematoxylin and eosin stain, mag. 10X).

Granulomas are characterized by the predominance of histiocytes that evolved into epithelioid cells, forming more or less defined aggregates that may contain various other inflammatory cells. The formation of granulomas is the result of a complex inflammatory interplay between a persistent non-degradable antigen and the host's chronic immune system of macrophage activity, Th1 cell response, B-cell overactivity, circulating immune complexes, and a vast array of biological mediators [1], [2]. Granulomas can be divided according to the inflammatory cells involved, their arrangement and the presence of associated features into necrotizing (caseous and suppurative) and non-necrotizing (tuberculoid, sarcoidal, xanthogranulomas, palisaded, foreign body etc.) [3], [4]. (Figure 1)

Cutaneous leishmaniasis (CL) displays a wide spectrum of clinical and microscopic findings that has been extensively described. Ridley et al. proposed grouping the microscopic manifestations into five groups. Group I represents an almost normal appearing skin biopsy with patches of collagen degeneration. Group II shows a predominant severe necrotizing process in the dermis. In Group III, the dermis is involved by a diffuse and heavy mixed inflammatory infiltrate. Group IV shows scattered Langhans giant cells and primitive epithelioid histiocytes. Well-formed granulomas and well-developed epithelioid histiocytes are prominent in Group V [5], [6], [7]. In contrast to cutaneous infections with Tuberculosis (TB) and other mycobacteria that are typically associated with necrotic granulomas [8], [9], [10], the type of granulomatous response defined in CL, group V, is tuberculoid in nature with exceedingly rare cases described with necrotizing granulomas [11], [12], [13]. Furthermore, Ridley modified parasitic index quantifies the parasitic load of amastigotes in cutaneous lesions and has a numerical score from 1 to 6 as displayed in table 1.

**Table 1:** Modified Ridley's parasitic index for quantification of amastigote load.

Parasitic index	Number of amastigotes per standard section
6+	≥100, 000
5+	≥10, 000
4+	≥1000
3+	≥100
2+	≥10
1+	≥1

The old world countries endemic for CL also happen to be endemic for other granulomatous diseases such as leprosy, tuberculosis and cutaneous mycoses. The most common diagnostic approach used in these countries is still microscopic examination despite the advances in molecular diagnostic techniques and culture methods [14], [15].

In our large cohort of 317 patients with molecularly proven diagnosis of CL, we encountered a significant number of caseating granulomas overlapping clinically and microscopically with the diagnostic picture of TB. This unusual finding can hamper the expedient diagnosis of CL in endemic and nonendemic regions especially with international travel and influx of immigrants from areas of the world where this parasite is nonendemic [16], [17]. Considering the implications of this new finding, we report the accurate incidence of caseating granulomas in CL and its related variables.

## Materials and Methods

### Case selection and clinicopathologic data

A search of the pathology and dermatology archives at the American University of Beirut Medical Center, Beirut, Lebanon; Tishreen University, Lattakia, Syria; Saad Specialist Hospital, Al Khobar, Kingdom of Saudi Arabia; and Shaukat Khanum Memorial Cancer Hospital and Research Centre, Lahore, Pakistan was performed. The search included patients with skin lesions diagnosed with CL between 1992 and 2010. Cases with sufficient clinical data and material for diagnosis confirmation by PCR were included. Cases with mucocutaneous leishmaniasis, visceral leishmaniasis and those who had received prior treatment were excluded. This study was approved by the American University of Beirut Institutional Review Board and the patient data used in this study was anonymized.

Formalin-fixed paraffin-embedded tissue blocks of 4.0-mm punch biopsies were obtained for each patient. Three hundred and seventeen skin biopsies from 317 patients (1 biopsy per patient) with CL in Lebanon (n = 47), Syria (n = 157), Saudi Arabia (n = 43), Pakistan (n = 66), Ethiopia (n = 2) and Iran (n = 2) were evaluated. Part of this material has been used in a previous publication by the same group [18], [19]. Clinical data collected included patient's age, gender, geographic location, eruption type (papule, nodule, verrucous or scar), duration and anatomic site. Microscopically, the hematoxylin and eosin stained sections were retrieved on all cases and reviewed by two pathologists (IK and JA). Multiple parameters were recorded including the modified Ridley's parasitic index (PI, Table 1),) [20] Ridley's pattern (RP) and the presence and the type of granuloma (Figure 1). Patients with immunosuppresion were excluded from the study. Despite the wide period of time (1992–2010), all cases were diagnosed and triaged in the same method.

### Molecular subspeciation

PCR confirmation was performed on all included cases with subsequent molecular subspeciation performed on all PCR-positive cases, following previously published protocol [21]. In brief, DNA was extracted from ribbons originating from the formalin-fixed paraffin-embedded tissue blocks. PCR assay was performed to amplify the *Leishmania* ribosomal internal transcribed spacer 1 (ITS1) using the primers LITSR (5′ -CTGGATCATTTTCCGATG-3′) and L5.8S (5′-TGATACCACTTATCGCACTT-3′). This was carried out using the VersoTM 1-Step ReddyMix Kit (Thermo Fisher Scientific Inc, Surrey, UK) in an amplification reaction of 50 µL. The Px2 thermal cycler (Thermo Electron Corporation, Waltham, Massachusetts, USA) was used for amplification under the following steps and conditions: 95°C for 2 min, 35 cycles of (95°C for 20 seconds, 53°C for 30 seconds, 72°C for 1 minute) and 72°C for 6 minutes. Following PCR amplification, digestion of the ITS1- PCR amplicons with restriction enzyme HaeIII was performed for restriction fragment length polymorphism (RFLP) analysis and consequent subspeciation. The ITS1 RFLP technique used allowed the identification of all clinically significant strains, including *Leishmania tropica*, *Leishmania major*, *Leishmania braziliensis*, *Leishmania donovani*, *Leishmania aethiopica* and *Leishmania infantum* [22].

The TB PCR test used for the detection of Mycobacterium DNA is the MTB/RIF assay (CEPHEID, Sunnyvale, USA), and performed according to manufacturers' instructions. The 3 specific primers in the *Xpert MTB/RIF* assay amplify a portion of the *rpoB* gene (accession number: AF057488.1) containing the 81 base pair “core” region. The 5 probes A, B, C, D, and E are able to differentiate between the conserved wild-type sequence and mutations in the core region that are associated with RIF resistance. This assay is performed on the GeneXpert platform by CEPHEID.

### Special stains

Special stains were conducted after deparaffinization and hydration with distilled water. GMS, PAS, AFB and GRAM stains were performed in the traditional way with standardized techniques [23], [24].

### Statistical analysis

Continuous variables were analyzed by *t*-test or Mann-Whitney rank sum test as appropriate.

Categorical variables were analyzed using chi-square test. A two-tailed p<0.05 was required for statistical significance.

## Results

### Clinical findings

The age of the patients recruited for this study ranges from 1 year old to 92 years old (median 24 years, SD = 21.23). The male gender was predominant (n = 175, 55.2%). The most common site involved was the head and neck (n = 145, 45.7%). Patients presented mainly with a plaque/nodular lesion (n = 156, 49.2%) followed by ulcer/verrucous lesions (n = 144, 45.4%). The duration of the lesion at biopsy time varied from 2 weeks to 132 months (median 5 months, SD = 10.6).

### Microscopic and molecular findings

Microscopic evaluation of the 317 cases of CL is illustrated in table 2. Sequencing showed a predominance of the leishmania tropica species in the majority of the studied 317 cases (n = 279, 88.0%) and in both groups of granuloma. Leishmania major was identified in the rest (n = 38, 11.9%). All cases were negative by MTB/RIF assay.

**Table 2:** The distribution of Ridley's pattern and modified Ridley's parasitic index among 317 cases with Cutaneous Leishmania.

Cutaneous Leishmania Cases (n = 317)			
		Number	Percentage
Ridley's Pattern	I	4	1.3
	II	28	8.8
	III	127	40.1
	IV	93	29.3
	V	65	20.5
Modified Ridley's Parasitic Index	0	115	36.3
	1	23	7.2
	2	17	5.4
	3	32	10.1
	4	56	17.7
	5	51	16.1
	6	23	7.2

### Clinicopathologic correlation

**Figure 2:**
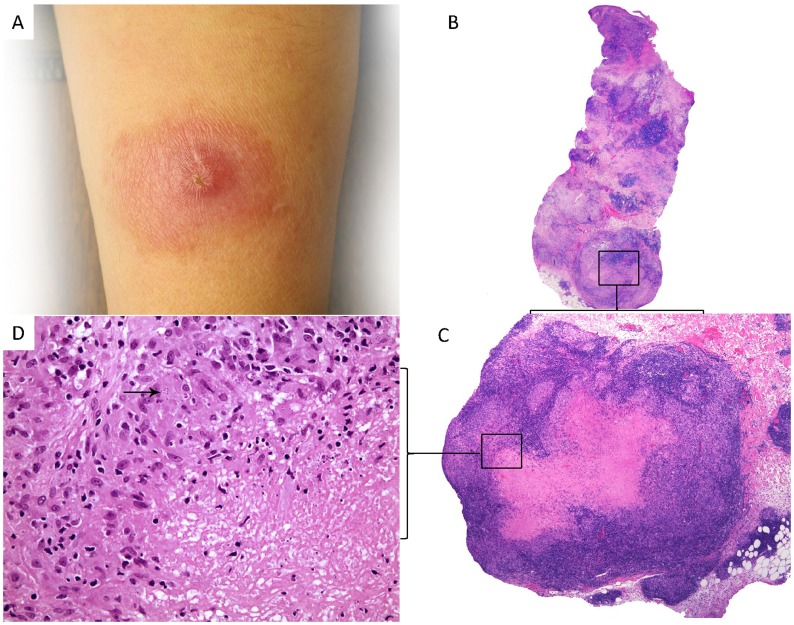
Cutaneous leishmaniasis presenting as an erythematous nodule (A) with superficial and deep nodular granulomatous dermatitis (B, hematoxylin and eosin stain, mag. 20X) exhibiting caseating granulomas (C, hematoxylin and eosin stain, mag. 40X) with identifiable amastigotes indicated by the arrow (D, hematoxylin and eosin stain, mag. 400X).

Out of the 317 cases there was a tuberculoid granuloma in 137 (43.2%) cases vs. a necrotizing granuloma in 58 (18.2%) cases including the 9 suppurative granuloma cases of CL. The majority of the cases with necrotizing granulomas were of caseating type (84.5%) the rest were suppurative in nature (Figure 2). For statistical purposes, cases with caseating and suppurative granulomas were lumped together as cases with necrotizing granulomas.

The only statistically significant clinical difference between the tuberculoid granuloma and the necrotizing granuloma cases was the geographic location. As such patients from Saudi Arabia exhibited a higher predominance of necrotizing granuloma (20.7%) than tuberculoid granuloma (8.0%). In contrast, patients from Syria showed a higher prevalence of tuberculoid granuloma cases (61.3%) than necrotizing granuloma cases (43.1%) with a p<0.0001.

For the microscopic features, the only statistically significant difference (p<0.0001) was the distribution of the RP where RP 2 and RP3 were more frequently encountered in cases with necrotizing granulomas (29.3% and 31%) vs. cases with tuberculoid granulomas(1.5% and 15.3%) respectively. RP 5 was mostly noted in association with tuberculoid granulomas (38.0%) than necrotizing granulomas (19.0%). The parasitic index variation between the two groups was not statistically significant (p = 0.09, see table 3).

**Table 3:** Comparison of clinical and microscopic variables between tuberculoid and necrotizing granulomas cases with geographic location and Ridley's pattern being the only 2 variables showing significant difference in distribution (ρ<0.0001).

		Tuberculoid granuloma (n = 137, 70.2%)	Necrotizing granulomas (n = 58, 29.8%)	p-value
Sex	Male	61.30%	60.30%	0.89
	Female	38.70%	39.70%	
Geographic location	Lebanon	16.10%	22.40%	<0.0001
	Syria	61.30%	43.10%	
	Saudi Arabia	8.00%	20.70%	
	Ethiopia	0.70%	0.00%	
	Pakistan	13.90%	13.80%	
	Iran	0.00%	0.00%	
Anatomic location	Head &Neck	53.30%	34.50%	0.11
	Upper Extremities	25.50%	36.20%	
	Lower Extremities	11.70%	24.10%	
	Other	0.70%	1.70%	
Lesion type	Papule	6.60%	3.40%	0.52
	Plaque/Nodule	53.30%	50.00%	
	Ulcer/Verrucous	38.70%	46.60%	
	Scar	1.50%	0.00%	
Ridley's pattern	1	0.70%	0.00%	<0.0001
	2	1.50%	29.30%	
	3	15.30%	31.00%	
	4	44.50%	20.70%	
	5	38.00%	19.00%	
Parasitic index	1	7.30%	6.90%	0.09
	2	3.60%	10.30%	
	3	8.80%	15.50%	
	4	21.90%	19.00%	
	5	19.70%	5.20%	
	6	5.80%	6.90%	
Molecular subtype	L. Tropica	93.40%	91.40%	0.61
	L. Major	6.60%	8.60%	

## Discussion

Leishmaniasis is a group of protozoan disease transmitted to human beings by the bite of female sandflies of the genera Phlebotomus in the old world and Lutzomyia in the new world [25]. CL is the most common form of Leishmaniasis and is endemic in more than 70 countries worldwide [15]. The diagnosis of CL is based on microscopic findings that seem to depend on the stage of evolution of the lesion. Early stage typical findings include a dense diffuse infiltrate of lymphocytes, plasma cells and histiocytes containing amastigotes throughout the dermis (RP 3–4). As the lesion progresses, tuberculoid granulomas with very few organisms if any impinge on the epidermis (RP 5) rendering the diagnosis more difficult [26].

The frequency of caseating granulomas in CL cases has been debatable in the literature.

Two studies identified caseating granulomas in association with CL. The first study performed by Boer et al. on a northern European population of 19 patients with CL identified granulomatous dermatitis in all cases. Nine of these granulomas were tuberculoid, five were sarcoidal and 2 were caseating. Sequencing of the following cases revealed all Leishmania to be L. infantum. In this study, 18 of 19 patients were misdiagnosed clinically and nine were also misdiagnosed histopathologically and included the atypical presentation of sarcoidal and necrotizing granulomas [17].

The second study included a larger cohort from Mexico and included 73 biopsies with localized CL. This study identified 21/73 (28.7%) cases of necrotizing granulomas vs. 32/73 (43%) cases of unorganized granulomas without necrosis. There was no relationship regarding the age and sex of the patient with the microscopic findings and the response to treatment. The responsible leishmania strain isolated was L. Mexicana [11].

On the other hand, the following 2 relatively large series showed no evidence of caseating granulomas in association with CL. Venkataram et al. described a series of 40 patients with CL from the Sultanate of Oman. Non-necrotizing granulomas were present in eight patients. The rest had no granulomas. As other parts of the Near East, leishmania tropica was the causing strain in this study [27]. In a larger series of 149 patients from Madrid during an outbreak of CL, the most encountered microscopic manifestation was non-necrotizing granulomas in 100 cases (67%) followed by a rich lymphohistiocytic infiltrate in 46 cases (31%). L. infantum was identified as the causative agent in 98% of the cases [25].

Our study represents the largest cohort of patients (n = 317) coming from different counties in the Near East region (Syria, Lebanon, Saudi Arabia, Ethiopia, Pakistan and Iran). The longest time to biopsy in our cohort, 132 months, compared to a maximum time of 42 months in the other discussed studies probably out of the socioeconomic status of the population and the provided medical care [25]. The high occurrence of necrotizing granulomas (18.2%) noted in our study has been rarely described in the literature except for a cohort of 71 patients from Mexico (28.7%) as previously mentioned. Moreover, our study of the Near East region relates some interesting variables to the occurrence of necrotizing granulomas that can raise the possibility of the diagnosing of CL. Especially that at the granulomatous stage of CL, identification of the amastigotes in the tissue can be very challenging which may lead the investigator toward other infectious diseases such as tuberculosis, leprosy and fungal infections.

Leishmania tropica has been reported to be the strain responsible for CL in the study coming from Oman which is the same strain recognized in 88% of our cases [27]. In addition, the dominant RP identified in our series are RP3 and RP4. Conversely, the most identified RP in the above discussed studies is granulomatous dermatitis (RP5) especially the tuberculoid type except for the study coming from Oman. The later study showed concordant RP with ours which happened to be the only study with the same causative strain “L. Tropica” as our study in contrast to L. Infantum and L. Mexicana identified in the other discussed studies. This can be explained by the adjunct geographical and close environmental factors between the Oman cohort and our cohort. These findings support the importance of the interaction of several factors related to the strain, vector and the host on the final clinicopathologic manifestation of the disease.

In our series, patients with caseating granulomas showed a slower healing process than patients with tuberculoid granulomas (6.2 months vs. 4.0 months) with no significant association with the specific strain or age group. This may be explained by inadequacy of the host immune response. In fact, studies have shown that necrotizing granulomas are due to the formation of immune complexes between antibodies and excess antigen in the center of the lesion. This setting develops when cell-mediated immunity, initially strong, begins to decline for unknown reasons, allowing the parasite to proliferate out of macrophage control. If cell-mediated immunity then improves again the number of microbes diminishes and immune complexes will form in antibody excess causing epithelioid granuloma formation instead of necrosis [2], [10]. This also explains the difficultly in identification of leishmania amastigotes during the granulomatous stage of the disease (late stage, RP5).

In conclusion, we document an 18.2% incidence of caseating granulomas in CL. Hence, CL should be part of the differential for cases with caseating granulomas in endemic regions in addition to TB and other causative infections. In addition, cases of CL with caseating granulomas also showed a slower healing process, with no association with specific species, which may be due to worse host immune response in such cases or to a more aggressive leishmania strains.

## Supporting Information

Checklist S1 STROBE checklist.
(DOC)
